# Conceptualizing the public good for genomics in the global South: a cross-disciplinary roundtable dialogue

**DOI:** 10.3389/fgene.2025.1523396

**Published:** 2025-07-02

**Authors:** Tayyaba Jiwani, Adjua Akinwumi, Jocelyn Cheé-Santiago, Yulia Egorova, Gabriel Frassetto, Ricardo di Lazzaro Filho, Iscia Lopes-Cendes, Mercedes Okumura, José Antonio Alonso Pavón, Alice B. Popejoy, David Skinner, Peter Wade, Matthias Wienroth, Ernesto Schwartz-Marin, Michel Satya Naslavsky

**Affiliations:** ^1^ Department of Sociology, Philosophy, and Anthropology, University of Exeter, Exeter, United Kingdom; ^2^ School of Communication, Simon Fraser University, Burnaby, BC, Canada; ^3^ School of Life Sciences, Arizona State University, Tempe, AZ, United States; ^4^ Department of Anthropology, University of Durham, Durham, United Kingdom; ^5^ Department of Genetics and Evolutionary Biology, Biosciences Institute, University of São Paulo, SãoPaulo, Brazil; ^6^ Dasa Genômica/Genera, Genômica, SãoPaulo, Brazil; ^7^ Department of Medical Genetics and Genomic Medicine, School of Medical Sciences; and Brazilian Institute of Neuroscience and Neurotechnology (BRAINN), University of Campinas (UNICAMP), Campinas, São Paulo, Brazil; ^8^ Faculty of Sciences, National Autonomous University of Mexico (UNAM), México City, Mexico; ^9^ Department of Public Health Sciences, University of California, Davis, United States; ^10^ School of Humanities and Social Sciences, Anglia Ruskin University, Cambridge, United Kingdom; ^11^ Department of Social Anthropology, University of Manchester, Manchester, United Kingdom; ^12^ School of Humanities and Social Sciences, Northumbria University, Newcastle-upon-Tyne, United Kingdom; ^13^ Hospital Israelita Albert Einstein, SãoPaulo, Brazil

**Keywords:** justice, genetic diversity, sovereignty, capitalism, inequality, governance, genomics, public-private

## Abstract

Since the Human Genome Project, initiatives to genetically sequence and profile populations around the world have expanded rapidly. The rationales guiding this expansion are diverse: on the one hand, the concentration of genetic technologies in the global North threatens to widen the yawning gaps in healthcare available in advanced versus developing nations. On the other, more ‘genetic diversity’ in global databases can reveal new points of genetic variation associated with health or disease. This promises to pave the way to a more personalized medicine of the future—more powerful and prosperous, with tailored prevention regimens and genetic treatments targeted to every individual’s specific genetic vulnerabilities. These rationales are advanced to claim a public good case for genomics. However, the expansion of genomics to underserved populations in the global South has provoked many sociopolitical and ethical challenges. Critics have pointed to the inevitable entanglement of genomics with private commercial interests. These concerns are overlaid on deeper anxieties stemming from global asymmetries in scientific and technological power, and historical patterns of value extraction from colonized and marginalized populations. How then do we disentangle the public good? How do we build a genomics science that is just and equitable for the vast majority of the world? This conversation convenes leading genomics practitioners and critical science studies scholars to address these questions. We draw on an ongoing transdisciplinary dialogue, integrating the natural and social sciences, and bring together perspectives and scholars from the global North and South. Our aim is to cultivate a more holistic and grounded engagement with the scientific and political challenges we face, to truly understand the requirements of a genomics that centers the question of justice.

## Introduction

An understanding of genomics as a “global public good” has motivated the spread of human genomics programmes to populations around the world (World Health Organization, 2002; Smith et al., 2004; Marsh et al., 2006). However, as noted by critics, the public good is an ambiguous concept, and genomics initiatives over the past 2 decades have struggled to live up to their claims. These initiatives and their outputs have neither been wholly “public” (but increasingly private and commercial), nor have they been received as “good” for all–in fact, they have even been accused of causing harm. How, then, do we understand the public good and shape an attendant genomic science?

A “global public good” is typically defined as a ‘good’ that is non-excludable and non-rivalrous (i.e., it is available for everyone and its use by some does not preclude its use by others) (Chadwick and Wilson, 2004). Central to the debate is how a good is defined and who gets to define it.

Mainstream genomics is guided by the assumption that it is fundamentally beneficial–it will advance scientific knowledge, improve healthcare, and drive the innovation-driven modern economy (Hardy et al., 2008; Séguin et al., 2008a). As such, global scientific and developmental bodies are preoccupied with bringing genomics to more populations in the world, to simultaneously ensure that no one gets ‘left behind’ in the coming “genomic revolution”, and to increase “genetic diversity” in global databases.

However noble their intentions, these projects have been strongly opposed, on the grounds that they do little to address structural socioeconomic and health inequalities and may in fact work to reproduce them (Reardon, 2005; Benjamin, 2015). Where populations across the Global South may provide their genetic samples and data for research, they will likely not be able to access or afford the resulting medical or technological benefits. These communities have thus challenged abstract and superficial narratives of diversity and inclusion in genomics, calling attention to structural inequalities and exclusions that must be addressed for genomics to be a global public good.

These critiques become more salient in view of the increasing commercial value of data, intellectual property and technological innovation resulting from genetic research (Sunder Rajan, 2006; Mitchell and Waldby, 2010). This value is currently overwhelmingly captured by pharmaceutical and biotech corporations based in the global North. Additionally, historical asymmetries in scientific, technological and economic power, alongside intellectual property regimes skewed in favour of the North, further impede the capacity for research and innovation in the global South.

These asymmetries–whereby the global South supplies raw data and materials that are capitalised for products and profit by the North–have unsurprisingly been characterised in neocolonial terms (Séguin et al., 2008b; Benjamin, 2009; Schwartz-Marín and Restrepo, 2013). For racialized and Indigenous populations, this pattern of resource extraction invokes histories of colonial exploitation and subjugation, which were also mediated via science and technology. They further challenge an idealised understanding of genomics as disinterested knowledge for “universal good”, pointing to the inequalities (re)produced by actually existing genomics.

As sociologist Steve Sturdy argues in his study of 20th century debates on genetic patenting, the idea of a “genetic commons” did not develop as a “neutral space of disinterested scientific research that naturally aligns with some abstract “public good”. Instead, it was “part of an innovation system that evolved to serve the interests of a range of stakeholders, among which the big pharmaceutical companies enjoy a dominant position” (Sturdy, 2025).

One strand of genomics policy has sought to address these challenges by insisting on free and open data sharing–preventing the privatisation of data from marginalised communities so that they can use it for their own benefit (Knoppers and Fecteau, 2003). However, unrestricted data access has raised complex concerns around personal privacy, bodily integrity, data sovereignty, and terms of data governance and stewardship. These debates have made it clear that open data sharing is not an adequate or universal solution to the inequalities that shape the terrain of genomics.

Nevertheless, policy prescriptions on genomics and the public good have overwhelmingly focused on the regulation of genomic practice, with emphasis on informed consent, materials and data sharing, or data privacy (Barker et al., 2013). The latter has been broadly construed to include “confidentiality, secrecy, anonymity, data protection, data security, fair information practices, decisional autonomy, and freedom from unwanted intrusion” (US Presidential Commission for the Study of Bioethical Issues, 2012). They have also argued for more participatory research and community-led data governance or stewardship to make the process of knowledge production safer and more ethical for the communities involved.

However, this focus on genetic practice takes for granted the claims of genomic knowledge as a good in and of itself. It overlooks a more fundamental examination of the disciplinary foundations (i.e., epistemology) of genomics.

Significant historical and sociological scholarship has now demonstrated that the frameworks and techniques of genetic analysis are not objective, neutral, or value-free. In fact, the development of genetic science through the 19th and 20th centuries was embedded in colonial agendas to racialize human difference and subjugate racialized populations deemed inferior (Burton and Ghoshal, 2024). Thus, continued genetic studies to classify, stratify and analyse populations–without challenging underlying scientific frameworks and techniques–risk perpetuating and entrenching ideas of racial difference, shaping racialised bodies, racialised technologies, and a racialised biomedicine.

In summary, defining a universal public good from genomic science is a complex undertaking. Genomic knowledge and innovation are refracted through existing structural inequalities–while working to reconstitute and reshape them–in ways that impede collective benefit for all, and even risk causing harm.

How then do we shape a genomics that centres justice? This roundtable convenes leading genomic scientists–from the public and private sectors–with critical social theorists to discuss this question. We draw on ongoing interdisciplinary dialogues, from the natural to social sciences, ethics and philosophy, with perspectives from the global North and South. Our aim is to present the multifaceted nature of the debate, introducing different disciplinary perspectives in conversational and accessible terms.

## Inclusion and representation under structural inequality

**Michel Naslasvsky:** Let’s start by discussing how we understand the public good in genomics. As you know, genomics studies—especially population genomics studies pertaining to disease and personalised medicine—lack population diversity, beyond European (and recently some Asian) populations. There are two main arguments for enhancing “genomic diversity”: first, to better map the risks of disease in different populations worldwide, enabling greater and more equitable access to genetic technologies. And second, to identify new genetic targets for therapeutic development, enter new markets, and collect valuable data. Broadly speaking, these arguments map onto tensions between the public good and private interest in contemporary genomics.

In Brazil, we recently built a DNA sequence bank. We didn't have enough funds for sequencing, so we partnered with an American company. The individual-level data is available for researchers on request, and we are exploring it to better understand the Brazilian population. This project is a win for public research, but there is pressure to increase the number of datasets, and the private sector seeks to retain rights to the data rather than allow public access. This is a concern for us; we want to better understand the debate on this issue.

**Ricardo di Lazzaro Filho:** In the past 10 years or so, genomics companies that managed to collect large numbers of genetic profiles grew very quickly in value. Gathering large amounts of data gave them a significant market advantage, including the ability to make new discoveries (e.g., new genetic associations for disease) and develop new products. However, this must be attached to a responsibility on them to contribute to scientific development in society. In my view, public availability of sequences is really important, especially from research that is done with public investment, by public universities and research institutes.

**Iscia Lopes-Cendes:** I don't have a problem with commercial enterprises dealing with genomics in Latin America. I have a problem when they aren’t transparent. Most such research in Latin America is publicly funded, with participants donating their genetic material for research. I don't think it's appropriate to use something acquired with public funding for economic exploitation. The data and results need to be publicly available.

Second, there is a lack of representation in genomics not just of geographic diversity, but speaking from the Latin American context, also of admixed populations. This creates methodological problems, for instance in genome-wide association studies (GWAS). I recently contributed data from admixed Brazilian patients to a consortium conducting a large-scale GWAS, but the data was ultimately excluded from analysis because it fell outside the project’s sharply defined population boundaries. So, the issue is more than just diversity–it’s also about developing new research methods that work for different populations.

**Ernesto Schwartz-Marin:** This made me think of the Mexican call for “genomic sovereignty” (Schwartz-Marín and Arellano-Méndez, 2012; Benjamin, 2015). The motivation for the so-called law of genomic sovereignty was precisely to stop the expropriation of “Mexico’s” genetic material by the global North. This expropriation was seen as reproducing global hierarchies and technological dependence – “we in Latin America provide the samples, while the results stay in the US or the global North”. Iscia clearly framed this issue–there is a “net gift” by people in Latin America, some of whom are Indigenous, who may never benefit from the genetic study they contribute to.

The issue of admixture was dealt with 15 years ago when the ‘Mexican genome’ was put forward by the Mexican Genome Diversity Project to represent the admixed Mexican population (Silva-Zolezzi et al., 2009). But I want to challenge this idea—do you think inclusion is inherently beneficial? Let’s imagine that admixture mapping makes it into the clinic. Is it enough just to have representation in the technology, when it can only be accessed by those that can afford the resulting healthcare? In Latin America and elsewhere, public money is financing investment in genomics (sometimes precisely in the name of diversity and representation) but ultimately, it’s setting us up for more social inequalities.

**Tayyaba Jiwani:** In the South Asian context, we’ve also seen the rhetoric that marginalized communities (minority castes, ethnicities, or indigenous groups) need more representation and inclusion in genomics. But in fact, such projects have ended up reinforcing their marginalization, because now they are contributing their biological material “in the national interest” – and even participating in clinical trials–while receiving few benefits in return. At the same time, their genomes are curated for international databases as “unique” pools of variation, further setting them up as populations for international scientific investigation (Sunder Rajan, 2010).

The definition of public good in genomics gets even trickier when you consider the commercial implications of drugs and technologies developed from new sources of genomic data. These technologies can end up benefiting a layer of national elites and entrepreneurs (Jiwani, 2023), who may lead the recruitment of marginalised communities to genomics initiatives, but ultimately would be the only ones benefiting from the research products, both economically and medically.

**Adjua Akinwumi:** One thing to note is the relationship of genomics with data. Conceiving of genomic data as a valuable resource supports a narrative of value extraction. Inclusion under this framework becomes a mechanism for extraction. We have seen this happen—in 2019, for example, Wellcome Sanger faced significant backlash for allegedly unethically commercialising DNA from an Indigenous group in southern Africa (Grens, 2019).

**Gabriel Frassetto:** Indigenous people have raised concerns about clinical genetics research and “gifting” access to their bodies. They also have concerns about data ownership and secondary use (reuse of collected data for later projects without consent). Their lack of trust stems from historical experiences when the results of such research negatively impacted these communities enormously. I think we must rethink how research is conducted–is it about a certain group, or is it for the group with the group. Are participants simply gifting samples and data, or are they included in the interests and agendas of the research?

## Rethinking governance and regulatory regimes

**Peter Wade:** We have so far been talking about global inequalities where samples/data from the global South are used to generate certain kinds of information by people from the global North, or to answer questions pertinent to people from the North. That’s one way of approaching the question of global public good.

Another, though related, question is about regulatory regimes: what are the ethics of the methods by which people generate data about their research participants? Margaret Sleeboom-Faulkner has written a lot about regulatory regimes in the global South, Asia and China in particular. She says there’s a widespread idea that regulatory regimes in some of these countries are rather lax, and this allows scientists greater leeway in generating knowledge or conducting certain experiments, clinical trials, and so on. She’s quite keen to kind of relativize that to the Chinese context.

Her argument is that “international norms” about bioethical procedures, which are basically Western norms, aren’t always applied that rigorously even in the West, as we know. And second, they shouldn't be applied in a blanket fashion to other regions of the world, where even if the scientists are aware of the norms (what she calls bioethical knowledge), they may not have the capacity or the political clout to effectively apply them. She argues that you have to look at how the norms become adapted to different regional “assemblages”, in which Western notions of ‘public good’ or ‘good practice’ may be seen quite differently (Sleeboom-Faulkner, 2014).

Having said that, we do need to bear in mind that some genomics projects can be carried out in, let’s say, authoritarian regimes where research participants’ rights are not respected, or in situations where it's difficult to get properly informed consent because participants might not be able to take on the kind of knowledge that is needed. All that is to say that we need to develop regulatory regimes that do not impose Western models in a blanket way.

**Ernesto Schwartz-Marin:** Peter, you’ve pointed to something I found fascinating during fieldwork in Colombia, Mexico, and other places–that we have very Eurocentric ideas of informed consent. I say Eurocentric because they are essentially copy-pasted from US and European ethics guidelines. Mexico’s national genomics program created its own version, in which they had Indigenous communities sitting in front of the scientists, doing a little bit of informed consent, talking a bit about Indigenous ontologies, though quite loosely (Séguin et al., 2008b). But at the end it amounted to signing away their genetic rights for the future of the larger ‘admixed’ national population (i.e., the Mestizos).

**Alice Popejoy:** The phrase public good in and of itself presumes two things: first, that “public” means everyone; and second, that the presumptive public good is viewed and experienced as good. I think that’s true in the case of genomics for some subsets of the population, especially those that have good access to all the structural and environmental factors required for one to be healthy.

Genetic heritability arguably matters more when you have all other detrimental factors in your environment taken care of, whereas when you don't have basic access to healthcare and preventive measures, then genetics becomes less relevant. So, the actual (or promised) value of genomics to populations that do not have the potential to benefit from genomic medicine, due to systemic injustices and inequality, is just not there. And thus, the “public good” in genomics is subjective, based on one’s positionality. It is rightfully not experienced as good when it is extractive and lacks a true value proposition.

We’re facing this in the Human Pangenome Reference Consortium (https://humanpangenome.org), which is based in the US but attempting to be a global resource. I’m part of the ELSI (ethical, legal, and social implications) team. We need to reconcile a lot of tensions in the consortium, a major one being the mandate to build an entirely open global resource with unrestricted data sharing, access, and use. All future uses of the data would be allowed based on a single consent transaction.

Barbara Koenig (also on the ELSI team) has written about informed consent as a transactional exchange being insufficient (Koenig, 2014), and that instead, consent to a governance process might be better and more ethical. I do not believe that a consent process involving a single transaction at one point in time, designed to cover all future uses of the resource is justified, considering that some of the methods that may be used to analyze and interpret this data have yet to be invented.

The main argument for creating an open resource with a broad future-use consent protocol is that everyone will have the opportunity to benefit from the data; in essence, its quality as a “public good” is equated with its unrestricted access and use. But that argument falls flat, as the benefits of it being entirely open are enjoyed mainly by highly skilled and trained computer scientists and bioinformaticians with access to rich resources, immense data storage and computational server capacity to deal with high-quality whole-genome sequence data—not just anybody and everybody.

This immediately reveals that such a resource can mainly be used by those who already have the means to leverage its potential, rendering it to the realm of the “private”, masquerading as a “public” good. Meanwhile, the potential risks of having such data freely available on the internet for anyone to use will fall squarely on those who have been recruited to donate their samples.

We (as the ELSI team or scientific investigators) don't necessarily need to define what the governance process for a reference resource should be a priori. But perhaps, when somebody provides their data for a resource-generating research project, they could be asked to consent to the co-development of a governance process for the stewardship of their data, and invited to be involved in designing it. I believe we can come up with nimble and grounded ways to do community-engaged genomics research, rather than thinking about better ways to articulate the value proposition the study investigators can offer in exchange for someone donating their blood or DNA for all future uses, forever.

## Public good for whom–negotiating access

**Adjua Akinwumi:** Given the ambiguity of the term public good, I wonder whether focusing on access might be more productive. We know that public good is linked with the question of access–public goods should be non-exclusive, i.e., everybody should have access. But if we think of the classic example of the road as a public good, we know that you need to have a car or motorcycle to access it. So I wonder if we instead centre the narrative on genomics and access–this could perhaps help us reflect on questions of benefit sharing, intellectual property, etc.

**Matthias Wienroth:** I want to add some conceptual food for thought. As Michael Warner argued, public goods are discursive elements—they call ideas into being and reflect specific desires and aspirations (Warner, 2002). That’s why my co-authored paper on genome editing in the United Kingdom links notions of the public good to “promissory discourses” – i.e., discourses promising a certain future (Wienroth and Scully, 2021). These reflect what is desirable, and implicitly, the “desirable publics”. So the discourse around public goods is in itself generative. It calls into being different types of publics–Warner’s “publics in particular” – and is linked to questions of access.

Thus, paying attention to who is negotiating what kind of public goods is important. Michael Callon wrote that the difference between public and private goods is in their rationales, their intended purposes. Again, this points to promissory discourses. What is a “private” versus “public” good? Well, it’s a question of what it promises and who gets access. Is it a zero-sum game? Then certainly it’s a private good. Do relatively large “deserving” publics have access, outside a zero-sum framework? Then it might be more of a public good.

But what’s important, as we’ve shown for genome editing, is that the discourses around these technologies–e.g., the claims around why we need more genetic information, more knowledge, etc., –delimit the public debate. They delineate what, why, and how we’re making these technologies and for whom. So, we want to, first, understand what these discourses look like, and then figure out how to challenge them.

**Yulia Egorova:** At the same time, there are legitimate questions around the promise of genetic medicine, as its quite far in the future, compared with existing medicine for example, and because marginalised communities already don’t have access. This means that genomics usually occupies a somewhat questionable, marginal place in different conceptualizations of the public good.

**Ricardo di Lazzaro Filho:** I would like to intervene from the perspective of running a commercial lab. Of course, I want genomics to be accessible for everyone, as do most commercial labs—they will have a larger market and greater profits. And of course, that’s a capitalist point of view. The reality is that the cost of DNA sequencing is declining. When we started offering ancestry tests here in Brazil 10 years ago, the price was more than three times higher. It's about 60 USD now, so still far from accessible for everyone in the country. But I think the lowering of cost is part of the technological process for most newly developed technologies.

Second, there can be practical, technical reasons why it can be hard to put products in the market that “work” for everyone and are broadly accessible. For example, we have a very admixed population in Brazil. When we use polygenic scores to calculate the risk of Type 2 diabetes, this works better in people with more European ancestry, because the score was developed from White Caucasian populations. It still works on people with less than 50% European ancestry, so we decided to put it on the market–but it’s difficult to define its accuracy when our ancestry is such a broad spectrum. So it’s hard for us to define the right ancestry cut-offs that ensure the accuracy of the test, which is one factor in making it accessible for all.

## Public good and the commercialisation of research

**David Skinner:** I believe we need more political economy to make sense of these issues. The life sciences have become intimately connected to contemporary capitalism. I’m repeatedly reminded of this because I’m based in Cambridge, United Kingdom, firmly in the global North, and am currently conducting a site-specific project about the Cambridge Biomedical Campus. This campus is the largest site of its kind in Europe and brings public and private sector science together in one place alongside a crucial third element, the National Health Service hospitals. AstraZeneca, the pharmaceutical company, is the second highest-valued company on the United Kingdom Stock Exchange. It has spent £1 billion moving its headquarters to the heart of this campus, footsteps away from the hospitals in which there are patients who offer a ready supply for clinical trials and research data.

When we talk about public or private good, we must recognize how the life sciences are inseparable from new forms of capitalism–what Kaushik Sunder Rajan has termed “biocapital” (Sunder Rajan, 2006). Yulia and Matthias quite rightly talked about the promise of the life sciences. This emphasis on “futures” extends to new forms of speculative capitalism.

You see this very acutely in the United Kingdom, where politicians portray the life sciences as providing the next big impetus to economic development. Equally, there are many scientists on the campus I am studying that began careers in academia but now believe that only the private sector can support the scale and ambition of their projects. This might help us rethink the public-private distinction.

**Ernesto Schwartz-Marin:** It’s not just promises about the future—we’ve called them “bioprophecies”, i.e., claims that we can intervene today to transform the future (Taylor-Alexander and Schwartz-Marín, 2013). These bioprophecies have always been at the public-private boundary. Public-private partnerships are embedded in these projects even in the very nationalist contexts of Latin America. In fact, they challenge the boundaries of what is “public”: they help funnel public resources into private hands, which is also why “genetic diversity data” is increasingly the raw material for private enterprises, as we see around the world. There’s an expropriation of public resources that is key for this political economy.

**Tayyaba Jiwani:** Indeed, the imbrication of the life sciences in contemporary capitalism must be central to our evaluation of public good claims. In fact, the discourse surrounding national genomics in multiple global South contexts has articulated a dual promise—a prosperous medical future and a prosperous economic future for the nation (Séguin et al., 2008a). The idea that genomics and biotech will help developing nations ‘catch up’ in economic terms with the West—it would help them build a modern “knowledge economy”, an innovation economy, and launch them into the bioeconomic age. It is very much an agenda of capitalist accumulation enacted by exploiting bodies in the global South and intertwined with global circuits of capitalist knowledge production for profit. That’s key to this discussion.

A related point is that increasing private commercial interest in the life sciences goes hand-in-hand with greater geneticization of disease itself. That is, a shift towards understanding disease in largely genetic and molecular terms (rather than say, complex interactions between genetic, systemic, social, and environmental factors). This, then, justifies the development of molecular treatments, diagnostics, etc., which have high commercial value as opposed to, say, public health interventions. So, disease also becomes a site of commodification and capital accumulation. And this ultimately lends to a much more commercialized health system, with issues of access that Adjua pointed out.

And again, we have to ask whether developing countries have the kind of financial capital and infrastructural capacity to realise this genomic medicine dream and its economic and medical promise for all.

**Matthias Wienroth:** Tayyaba’s point about the geneticization of health and illness is closely linked to techno-solutionism, the idea that if we invest in technologies, we will find solutions. This belief might make us forget about alternative solutions, or necessary investment into existing solutions. It in itself has promissory, almost self-fulfilling effects, in terms of what infrastructures, research agendas, or relationships we invest in at the moment, and which ones we don't prioritize.

**Adjua Akinwumi:** This point about disease commodification is very important. It points to Peter Conrad’s extensive work on “medicalization”, which describes how conditions and behaviors are transformed into medical issues, often to serve the interests of pharmaceutical companies which pathologize these issues to market new drugs (Conrad, 2005).

Interestingly, in the same year that Conrad is writing about the shifting engines of medicalization, we have the drug BiDil approved by the FDA. This drug was specifically created for, and marketed to, African Americans, based on data suggesting they were more susceptible to heart disease than other racial groups. This effectively created a market for race-based drugs, which has proven to be controversial for many reasons, chiefly that it obscures the broader socio-political factors leading to health disparities, offering a seemingly simple solution to a complex issue. Jonathan Kahn has written an insightful book, Race in a Bottle, on this issue (Kahn, 2014).

**Matthias Wienroth:** Continuing our conversation on knowledge economies and nation-building–Arguments for the development of genomics as “programs of state” are based on the idea that the nation will help advance and benefit from such initiatives. We’ve especially seen in this in the United Kingdom through narratives of the bioeconomy as the neoliberal “next step”. There’s an implied obligation in these narratives on citizens to participate.

This implied obligation has been taken even further, by building data platforms and networks where companies such as Google are given access to data. Even Palantir, a private spy company from the United States, is building a data sharing platform for the NHS in the United Kingdom. So, private goods are being rendered as public goods, even public obligations. This makes it even more difficult to talk about public-private distinctions.

Rafaela Granja and I have been writing about governance in an emerging assemblage of practice in forensic genetics (Wienroth and Granja, 2024). Specifically, about the dissolving of boundaries between different key players—commercial, academic, and state players—in the genetics domain as their interests overlap. While disconcerting for something as vital to society as criminal justice and its fair and impartial governance, similar developments have occurred elsewhere, e.g., we already know from the field of medical devices that industry interests basically give direction on how to govern this area. There is a strong alignment of thinking around public goods with commercial industrial interests, and the problem of governance and accountability remains significant.

**David Skinner:** I also think that so much of the ethical debate about genetics is framed in terms of sharing or not sharing data—how you collect data, how you keep it, how you share it, or should you share it. This is obviously important, but there are other kinds of flows and blockages that are also important, given our discussion about global dynamics and public/private good—the sharing or not of expertise, techniques, and technologies associated with genomics, for example,.

## Genomics and the risk of harm

**Ernesto Schwartz-Marin:** It seems to me that much of the debate is around inclusion and informed consent, but maybe there are some reflections on the inherent injustices of genomic research as well? I think genomics reproduces colonial dynamics in so many ways, not only through colonial racial definitions, but also through colonial structures of power and political economy, its obsessions with data, and this politics of inclusion that is couched in altruism but is in fact extractive from minority populations. This is a huge issue that shapes the debate for me (Schwartz-Marín and Restrepo, 2013).

One of my colleagues, an Indigenous scientist, in Ecuador sent samples to a collaborating lab in Germany studying a disease. He’s been waiting for 3 years to receive the results. Meanwhile, the German lab has used the samples, published papers etc., while he was only able to visit them for a week to receive some training. This is clearly a colonial relationship.

Moreover, there’s a process of endo-colonialism—when genomic scientists in Latin America collect samples, they promise that genomics is going to bring lots of health benefits, that by knowing our DNA we can change the future course of a very impoverished nation. But again, those promises only hold true for a small section of the population.

And really, I don't think our tools and frameworks for genomics are fit for purpose. For example, how can we use liberal notions of property and individual rights to govern genomics, when DNA is personal, familial, communal, all at the same time? It challenges our legal paradigms and turns on its head the way we think about responsibility and even public good.

**Alice Popejoy:** Speaking of colonial racial definitions, the 1,000 Genomes Project explored new governance mechanisms and community-engaged genomic practices by establishing committees of community members to advise the project on how their data should be described in public repositories and published research. Unfortunately, at some point, the data from 26 different population groups were lumped together into five continent-level “super-population” groups that roughly coincide with U.S. racial/ethnic categories. It seems this made it easier for those using the data to conduct stratified analyses, thereby erasing the diverse architecture of the resource and ignoring community consent processes.

Suffice to say, the biocolonialist extractionism of the global North versus the South is thriving and gaining momentum in the US, as a system built on racial capitalism, and I think it would be a defensible argument to say that genomics is inherently unethical in its current form.

**Jocelyn Cheá-Santiago:** In Mexico, the idea of genomics as public good has also at times legitimized unethical ways to obtain data. Some private companies are developing DNA tests in Mexico, and we don't know how they get their data, or conduct their protocols, or which researchers are involved and whether they have the required expertise.

**José Alonso:** Companies like SOMOS or Codigo46 are offering direct-to-consumer genetic tests, providing information about ancestry and health risks, but it’s unclear who they are, what databases they are using to compare and analyse their samples, and how they are returning results.

**Matthias Wienroth:** In policing, we have this wonderful term called “noble cause corruption” (Caldero et al., 2018). We’ve coined a similar term for forensic genetics – “noble cause casuistry”, which can also apply to genetic research to some degree (Wienroth and McCartney, 2024). It refers to the claim made by forensic actors: “some of our work has delivered great goods that have been recognised, and as such, all the work we do in the same direction is of the same ethical value”. So there’s an implicit building of an “ethical regime” – the notion that Emma Kowal and Joanna Radin developed, and that Jackie Leach Scully and I applied in our discussion on “promissory ethical regimes” (Radin and Kowal, 2015; Wienroth and Scully, 2021). Forensic geneticists will say that their work is delivering criminal justice, so it's for “good”. However, this can at times lead to, and seemingly rationalise, dubious ethical practices in policing and forensics.

## Building a just genomics

**Michel Naslavsky:** Our discussion makes it clear that there’s a tension between data collection and sharing, versus data protection or limiting data exploration. For technological development, there is a need for data from more people integrated into research. But this raises fears of being exploited, of reviving colonialism in a bio-capitalist economy that we know is booming. So, what are the potential ways forward? How do we understand the needs of marginalised groups and help them participate in genomics programs while ensuring that they receive the benefits? Should we demand that companies reward people that share their data?

**Ernesto Schwartz-Marin:** I think that genomics is too obsessed about increasing genetic “diversity” – I can understand why, because you want to make sure that you cover people with different genetic backgrounds. But we need to reflect more on health systems and the neoliberal politics they’re embedded in, which are producing and reproducing (social and health) inequalities.

Many countries in the global South that have the money to invest in genomics, like Mexico or Brazil, have very neoliberal healthcare systems. Mexico has a public healthcare system, but in practice it doesn't work. There’s a two-tier health system—one tier exists in a ‘sci-fi world’ where people can have genomic tests, and the other lacks even basic health facilities. DNA for genomics is obtained from the latter, just to even set baselines for the first. To understand admixture in the national population, you get DNA from Indigenous people. This extractivism is the biggest question for me when it comes to justice and the public good.

**Ricardo di Lazzaro Filho:** In terms of giving donors a return, either financially or in another form—I’ve been following the market as part of a genomics company for the last 15 years and while a couple of initiatives tried this, I don’t think they figured it out. It's hard to develop a system that is fair, accounts for the complexity of genomics, and really works. In Brazil as in many other countries, we cannot legally pay someone for their samples or for participating in a study, which also makes this difficult.

**Ernesto Schwartz-Marin:** I agree that we should not pay people for their DNA, for a simple reason–we don't know its value. The value is currently speculative, promissory; it’s like an asset. And our payments would reinforce the capitalist understanding of DNA as an avenue for value extraction, which is a notion we have been critiquing.

Some people argue for benefit-sharing, but what does that mean concretely? I was contacted by an ancestry company in Mexico called SOMOS. They wanted to make a virtual “DNA wallet”, allowing you to “invest” your DNA in different projects. I thought that’s better than the NHS gifting my data to Google, but of course, this project is really only for “Whitexicans” as we call them—posh White Mexicans that can engage in this economy. And again, it doesn't address the expropriation of samples from marginalized communities.

Actually, we don’t realise how much existing regulatory frameworks for databases are shaped by our understanding of value and exchange, or our obsession with data—all shaped by capitalist economic frameworks. So, if you ask me, the first thing we need to do is unlearn. For example, it would be great if we can talk about what a Zapotec genome database would look like–not a database about Zapotecs, but one which foregrounds the values of different Zapotec communities.

I’m also against the notion of “sovereignty”, whether Indigenous or national, because it enables authoritarian rule and elite-centric approaches to governance. But I have no idea what the alternatives are. I’ve tried adopting governance mechanisms that are more bottom-up in the databases I’ve built, and they’re as messy as the elite ones, if not more. This is a huge challenge, but we need to face it if we really want to tackle the issue of justice in genomics.

**Mercedes Okumura:** Addressing Ernesto’s comment, maybe we should go back to the basics and remember that one of the three elements of ethics is the intrinsic good. How can we bring back this duality between instrumental and intrinsic value? It’s very important to understand how different stakeholders see this. Also, as someone who works with both past and present human populations, there is very robust discussion among bioarchaeologists about inter-generational ethical issues. This requires expanding our focus from problems in the present to building a bridge between generations.

**Michel Naslavsky:** There’s another aspect in the economic argument to consider—that of intellectual property. I recently spoke about this with Nicola Blackwood, who leads Genomics England, a public corporation governed by the Department of Health. They have also received some private seed funding, and the question of intellectual property is still quite open. Intellectual property frameworks are often advanced as an avenue to protect the public good, but there is active debate on how to govern its ownership, privatisation, and financial returns.

**Alice Popejoy:** Based on my experience consulting for Genomics England in the initial stages, I understand their goal was to help the NHS figure out how to create and roll out a genomic medicine program within the NHS public healthcare system. As such, Genomics England was at least trying to build a public health genomics project. And that is, in essence, a public good because access to its services, and the potential to benefit from its findings, isn't restricted a priori by one’s ability to pay for healthcare, unlike in the US.

Such projects may be less defensible as public good when they have no clear path to generating shared value from whatever is being produced or learned. Or if they’re creating data accessible by commercial entities, who have no obligation to share profits with the people whose data they used.

**Tayyaba Jiwani:** I echo what others have said about payment not being an adequate return, because it reinforces the notion of DNA as personal “property”, as something that can be commodified, which is problematic.

Second, of course it's very difficult to address the challenges we’ve discussed. But I think one entry point could be to evaluate the various national genome programs that were established around the world as ‘public health genomics’ programs, and ask to what extent they have been successful in meeting their goals. To my knowledge, these programs set up in “emerging economies” (like Mexico or India) are the biggest and most prominent “public good” initiatives in genomics. They aimed, for example, to find DNA markers associated with the risks of various diseases to build preventive health programs or to find markers associated with drug or vaccine resistance.

And now that some of these programs a couple decades old, it would be a good exercise to examine their initial gains. Have they delivered on their public good aims? Are their benefits, such as they are, publicly accessible or mainly privatised and exclusive? And if they didn't achieve the public good, where did they go wrong? And therefore, are such programs a realistic goal for health systems in other developing countries?

**Ricardo di Lazzaro Filho:** I don't know if someone has done the math on our investments in genomics versus what they’ve brought to society. Equally, I think the complexity of human genetics adds to the time it’s taking to see its benefits. But also, I believe we have had a lot of indirect benefits from this knowledge, e.g., precision treatments for cancer that are bringing more years to patients. More new drugs are being developed with the help of genomics. Most of us probably received an mRNA vaccine against COVID-19, and these advances in oligotherapy were at least partially enabled by the field of genomics. So, it's hard to measure, but I think we are receiving a lot of benefits, though I understand that the promises were huge in comparison.

**Peter Wade:** I want to take up Tayyaba’s suggestion. If we take the Mexican Genome project, which published some of their results (like the key paper, Silva-Zolezzi et al., 2009), my impression is there’s nothing to show for it really, in terms of public health at the moment. Or Mike Fortun’s book Promising Genomics about Iceland, when their national project had been going for some time (Fortun, 2008). Do we have anything to show for that? My sense is that there hasn't been a big payoff yet. But then, the promissory nature of this science means that’s the whole point; you can defer it infinitely into the future.

**Ernesto Schwartz-Marin:** I echo Tayyaba’s idea; that’s a great way to think about public good. Let’s see what has happened 20 years on. What sorts of promises were delivered? In the Mexican national program, led by the Genomic Institute of Medicine (INMEGEN), they built a whole department for “translational genomic medicine”, which was to use all the knowledge from the genome project to build start-ups and inject them into the economy. But little has happened.

One of their aims was to reduce diabetes, but Mexico is still one of the top countries in the world for diabetes prevalence. They claimed, back when they launched in 2004, that they will reduce 25% of diabetes incidence in the country by 2025. They received 120 million USD in public funds for genetic studies and interventions towards this goal, even though we know that there are significant social and environmental factors contributing to diabetes. So we can start testing whether they delivered on this promise.

This would be a very interesting way to ground the political economy of genomics and databases. We have lots of theory about promissory discourses, or “assetization” versus commodification of genomics, but now we’re in a position to do more empirical research. And maybe that’s how we can start rethinking the instruments and frameworks of ethics and governance as well.

**Yulia Egorova:** Around 10 years ago, when I was conducting research in India, with geneticists participating in national genomics initiatives, I asked them this exactly—when are we going to see the benefits promised? And the answers would be 30, 40, 50 years’ time, or maybe 100 years. So, they were quite honest about it. It will be very interesting to see what has come from that research, and whether communities or the healthcare system have seen any benefits.

**Tayyaba Jiwani:** This has been a wide-ranging conversation with much food for thought. It’s clear the public good case for genomics is not straightforward, when you place it in the context of global political, economic, and scientific hierarchies. We need to re-examine the claims and promises of the science, and its refashioning of our political economies, in ways that will involve more sustained dialogues across disciplines and geographies. This discussion was just a first step in that direction.

Thank you, everyone, for participating.

## Bios

Tayyaba Jiwani is a research fellow at the University of Exeter (United Kingdom). She has a background in molecular genetics and is now studying the history and political economy of genomics in South Asia.

José (Pepe) Antonio Alonso Pavón is a PhD candidate in Biological Sciences, specialising in bioethics and social studies of science, at the National Autonomous University of Mexico (UNAM). Currently he co-coordinates the Students’ Community at UNAM’s Interdisciplinary Seminar on Racism and Xenophobia.

Adjua Akinwumi is a PhD candidate at Simon Fraser University (Canada) and Mellon Data Fluencies Fellow at the Digital Democracies Institute. She works on issues related to health risk and emergency big data technologies.

Jocelyn Cheé-Santiago belongs to the Binnizá people of the Isthmus of Tehuantepec, Oaxaca. She is a PhD student in Biology and Society at Arizona State University and is interested in research on the governance of indigenous peoples’ genomic data, the laws and international treaties on Indigenous Peoples’ rights and how they intersect with scientific practice.

Yulia Egorova is Professor in anthropology at Durham University (United Kingdom), and Director of the Centre for the Study of Jewish Culture, Society, and Politics. She has extensively studied the anthropology of science and religion, specifically the responses of religio-cultural communities to population genetics.

Gabriel Frassetto is a PhD Candidate at the Biosciences Institute of the University of São Paulo (Brazil), as a member of the Laboratory of Human Evolutionary Studies. His research is focused on the ethical implications of ancient DNA studies, particularly in South American contexts.

Ricardo di Lazzaro Filho is a medical doctor with a masters in genetic counselling and a doctorate in human genetics. He is a co-founder and former CEO of Genera, the leading consumer genetics laboratory in Latin America. The company currently operates in Brazil, Argentina, Uruguay, and Chile.

Iscia Lopes-Cendes is a physician-geneticist, and professor of medical genetics and genomic medicine at the University of Campinas (UNICAMP). Her research focuses on neurogenetics. She founded the Brazilian Initiative on Precision Medicine, Latin America’s first public genomic database, and contributed to the development of LatinGen, a platform for sharing genetic and genomic information in the region.

Mercedes Okumura is a bioarchaeologist specialising in ancient DNA. She is an associate professor in genetics and evolutionary biology at the Biosciences Institute of the University of São Paulo (Brazil), and head of the Laboratory for Human Evolutionary Studies.

Alice B. Popejoy is an assistant professor in the Department of Public Health Sciences (Epidemiology Division) at the University of California, Davis, and an affiliate member of the UC Davis Comprehensive Cancer Center and the Aggie Square Advancing Health Equity program.

David Skinner is a sociologist based at Anglia Ruskin University (United Kingdom). He has extensively studied the intersection of race and science, as well as forensics and the management of DNA databases.

Peter Wade is Professor in social anthropology at the University of Manchester (United Kingdom). His research has extensively explored the intersection of genetic knowledge with ideas about nation, race, and gender in Latin America.

Matthias Wienroth is an assistant professor at the Northumbria University (United Kingdom) Centre for Crime and Policing. He studies the social, ethical, governance, and knowledge aspects of biotechnologies and biometrics for forensic and surveillance purposes.

Ernesto Schwartz-Marin is an assistant professor in anthropology at the University of Exeter (United Kingdom). He is a science and technology studies (STS) scholar specialising in the study of biomedicine, forensics, and citizen science.

Michel Satya Naslavsky is an assistant professor in genetics at the University of São Paulo (Brazil) and a researcher at the Hospital Israelita Albert Einstein. His research is focused on genomic admixture effects on rare and common phenotypes, medical genetics, and bioethics.
